# Treatment cost assessment for COVID-19 inpatients in Shenzhen, China 2020–2021: facts and suggestions

**DOI:** 10.3389/fpubh.2023.1066694

**Published:** 2023-05-04

**Authors:** Shasha Yuan, Ting Li, Cordia Chu, Xiaowan Wang, Lei Liu

**Affiliations:** ^1^Institute of Medical Information & Library, Chinese Academy of Medical Sciences & Peking Union Medical College, Beijing, China; ^2^National Clinical Research Centre for Infectious Diseases, The Third People's Hospital of Shenzhen, Shenzhen, China; ^3^Centre for Environment and Population Health, School of Medicine and Dentistry, Griffith University, Brisbane, QLD, Australia

**Keywords:** COVID-19, inpatient treatment cost, cost components, clinical classification, China

## Abstract

**Background:**

Knowledge regarding the treatment cost of coronavirus disease 2019 (COVID-19) in the real world is vital for disease burden forecasts and health resources planning. However, it is greatly hindered by obtaining reliable cost data from actual patients. To address this knowledge gap, this study aims to estimate the treatment cost and specific cost components for COVID-19 inpatients in Shenzhen city, China in 2020–2021.

**Methods:**

It is a 2 years' cross-sectional study. The de-identified discharge claims were collected from the hospital information system (HIS) of COVID-19 designated hospital in Shenzhen, China. One thousand three hundred ninety-eight inpatients with a discharge diagnosis for COVID-19 from January 10, 2020 (the first COVID-19 case admitted in the hospital in Shenzhen) to December 31, 2021. A comparison was made of treatment cost and cost components of COVID-19 inpatients among seven COVID-19 clinical classifications (asymptomatic, mild, moderate, severe, critical, convalescent and re-positive cases) and three admission stages (divided by the implementation of different treatment guidelines). The multi-variable linear regression models were used to conduct the analysis.

**Results:**

The treatment cost for included COVID-19 inpatients was USD 3,328.8. The number of convalescent cases accounted for the largest proportion of all COVID-19 inpatients (42.7%). The severe and critical cases incurred more than 40% of treatment cost on western medicine, while the other five COVID-19 clinical classifications spent the largest proportion (32%−51%) on lab testing. Compared with asymptomatic cases, significant increases of treatment cost were observed in mild cases (by 30.0%), moderate cases (by 49.2%), severe cases (by 228.7%) and critical cases (by 680.7%), while reductions were shown in re-positive cases (by 43.1%) and convalescent cases (by 38.6%). The decreasing trend of treatment cost was observed during the latter two stages by 7.6 and 17.9%, respectively.

**Conclusions:**

Our findings identified the difference of inpatient treatment cost across seven COVID-19 clinical classifications and the changes at three admission stages. It is highly suggestive to inform the financial burden experienced by the health insurance fund and the Government, to emphasize the rational use of lab tests and western medicine in the COVID-19 treatment guideline, and to design suitable treatment and control policy for convalescent cases.

## 1. Introduction

Coronavirus disease 2019 (COVID-19) was first reported to the World Health Organization (WHO) at the end of 2019 (1). It has rapidly become the worst global pandemic in a century (2). The clinical presentation of COVID-19 ranges from mild to severe symptoms, including fever, cough, shortness of breath, and lung infections, and it results in high morbidity and mortality (1). Hospitalizations for clinical treatment of COVID-19 were more costly compared to those for treatment of acute respiratory failure and pneumonia or influenza (3). The high prevalence rate of COVID-19 has imposed a heavy economic burden on healthcare system directly that may result in rationing or painful cost-control approaches (4).

Knowledge of treatment cost for COVID-19 inpatients in the real word is greatly needed to better improve the COVID-19 treatment guidelines and to inform scarce financial health resource planning, in particular for health insurance fund arrangements and public finance budget decisions (5, 6). Yet, few published studies have focused on examining the COVID-19 treatment cost by using data derived from actual COVID-19 patients, and most of those are from the United States of America (USA) (5, 7–9). Relatively more evidence has concentrated on the analysis of inpatient characteristics and clinical outcomes of COVID-19 inpatients by now (5, 8). Due to lack of actual treatment cost data at the micro level, studies regarding the economic evaluation of COVID-19-related interventions have had to use cost inputs from other diseases (such as pneumonia and influenza) (10, 11), or official data at the macro level (12), which would lead to estimation bias.

In China, the treatment cost for COVID-19 inpatients is covered by China Health Insurance Fund and public finance for the Chinese population. COVID-19 inpatients pay no out-of-pocket costs, which has ensured timely treatment of infected patients but also has resulted in huge financial burden for the Government and China Health Insurance Fund (13). Currently, the payment system for COVID-19 inpatients is primarily based on fee-for-service. The inpatient treatment cost is composed of different cost types: medicine (western and traditional medicine) cost, lab testing cost, medical imaging cost, beds cost, consultation cost, nursing cost, medical therapy cost, medical materials cost and surgical cost, as called “cost components” in this study. Understanding the level and distribution of treatment cost associated with COVID-19 inpatients is critical for both patients and policymakers. However, only a few studies have been found to describe the distribution of COVID-19 inpatient treatment cost (14–16). The study periods were all in early 2020 (the initial stage of COVID-19 pandemic) and the samples were <120 patients. The changes of treatment cost for COVID-19 inpatients in a longer period remains unclear. Moreover, we also know little about the difference of inpatient treatment cost (particularly about specific cost components) among different COVID-19 clinical classifications. The knowledge gap needs to be filled urgently.

Furthermore, clinical guidelines have been issued and implemented by WHO and most countries to guide the treatment of COVID-19 inpatients in clinical practice. In China, the first version of COVID-19 treatment guideline was released in January 2020 by the National Health Commission (NHC). Generally, the COVID-19 treatment guidelines summarized the etiological characteristics, epidemiological characteristics, pathological changes, clinical features, diagnosis, clinical classification, population with high risk of severe/critical illnesses, early warning predictors for severe/critical illnesses, differential diagnosis, case identification and reporting, treatment, nursing, discharge criteria and precautions after discharge, patient transfer, control of nosocomial infection in medical institutions, and disease prevention. China National Health Commission and the National Administration of Traditional Chinese Medicine convened a group of experts to timely revise the relevant content of the guidelines (18). During the study period of 2020–2021, eight versions of treatment guidelines have been released in China. Due to a lack of standard clinical treatment for COVID-19 inpatients, these guidelines play a dominant role to guide the physicians in clinical practice, which would impact the utilization of medical resources and then influence the treatment cost and cost components of COVID-19 inpatients. In this regard, it is meaningful and necessary to explore the changes of treatment cost components of COVID-19 inpatients at different stages of treatment guidelines to further examine the utilization of medical resources in treating COVID-19 inpatients.

This study seeks to comprehensively analyze the treatment cost for COVID-19 inpatients in Shenzhen, China in 2020–2021. To this end, we aim to analyze the total treatment cost and specific cost components for COVID-19 inpatients and how they are distributed across different COVID-19 clinical classifications, at different admission stages. Our findings can address the knowledge gap of treatment cost for COVID-19 inpatients in China and provide important information for future economic evaluations of interventions for COVID-19 and to form better COVID-19 treatment guidelines.

## 2. Materials and methods

### 2.1. Data collection

We used de-identified discharge claims from the hospital information system (HIS) of COVID-19 designated hospital in Shenzhen City. This hospital is a tertiary level infectious disease hospital and receives all the confirmed COVID-19 cases in Shenzhen during the pandemic period. At this time, it is the best available data from real-life COVID-19 inpatients.

Data regarding inpatient demographics (age, sex, insurance status), hospitalization characteristics (time of admission and discharge, LOS, comorbidity, COVID-19 clinical classifications), and treatment cost including different cost components were collected from the HIS. The data were all de-identified and was performed in accordance with the Helsinki Declaration.

The inclusion criteria were (a) hospitalizations with a discharge diagnosis for COVID-19 from January 10, 2020 (the first COVID-19 case admitted in the hospital in Shenzhen) to December 31, 2021 (*n* = 1,799); (b) clear COVID-19 clinical classifications (asymptomatic, mild, moderate, severe, critical, convalescent and re-positive; excluding 371 records); and (c) complete information records regarding treatment cost and patients' socio-demographic information (excluding 30 records). Finally, 1,398 inpatients were included in this study. The sampling diagram is shown in Supplementary Figure S1.

### 2.2. Cost components

As aforementioned, the fee-for-service payment system for COVID-19 inpatients is adopted in China (16). Therefore, the inpatient cost is closely related with fee items. There are mainly nine cost types (16, 17): medicine cost, lab testing cost (referring to check a sample of blood, urine, or body tissues), medical imaging cost (referring to produce detailed images of the body using forms of energy, such as X-ray, soundwaves, magnetic fields, and radioactive substances), beds cost, consultation cost, nursing cost, medical therapy cost (referring to medical therapy process by doctors and nurses such as supplemental oxygen, salvage, intravenous injection, blood collection, etc.), medical materials cost and surgical cost. They are called “cost components” in the following analysis.

### 2.3. The categorization of COVID-19 inpatients

#### 2.3.1. The COVID-19 clinical classifications

Based on China diagnosis and treatment guidelines (18, 19) and clinical practices of COVID-19 treatments, the COVID-19 inpatients were categorized by seven clinical classifications: asymptomatic, mild, moderate, severe, critical, convalescent and re-positive cases. The classification was performed by the doctors and cross-validated by the expert group consist of doctors and management staff in the hospital every day. The characteristics of each clinical classification are shown in Supplementary Table S1.

In this study, we particularly added the analysis of treatment cost for asymptomatic cases and convalescent cases, which were two special classifications categorized in China's clinical practices that have rarely been seen in other studies. Therefore, we are able to present comprehensive cost analysis regarding COVID-19 inpatients in China.

#### 2.3.2. The division of admission stages by different clinical treatment guidelines

**Figure 1:**
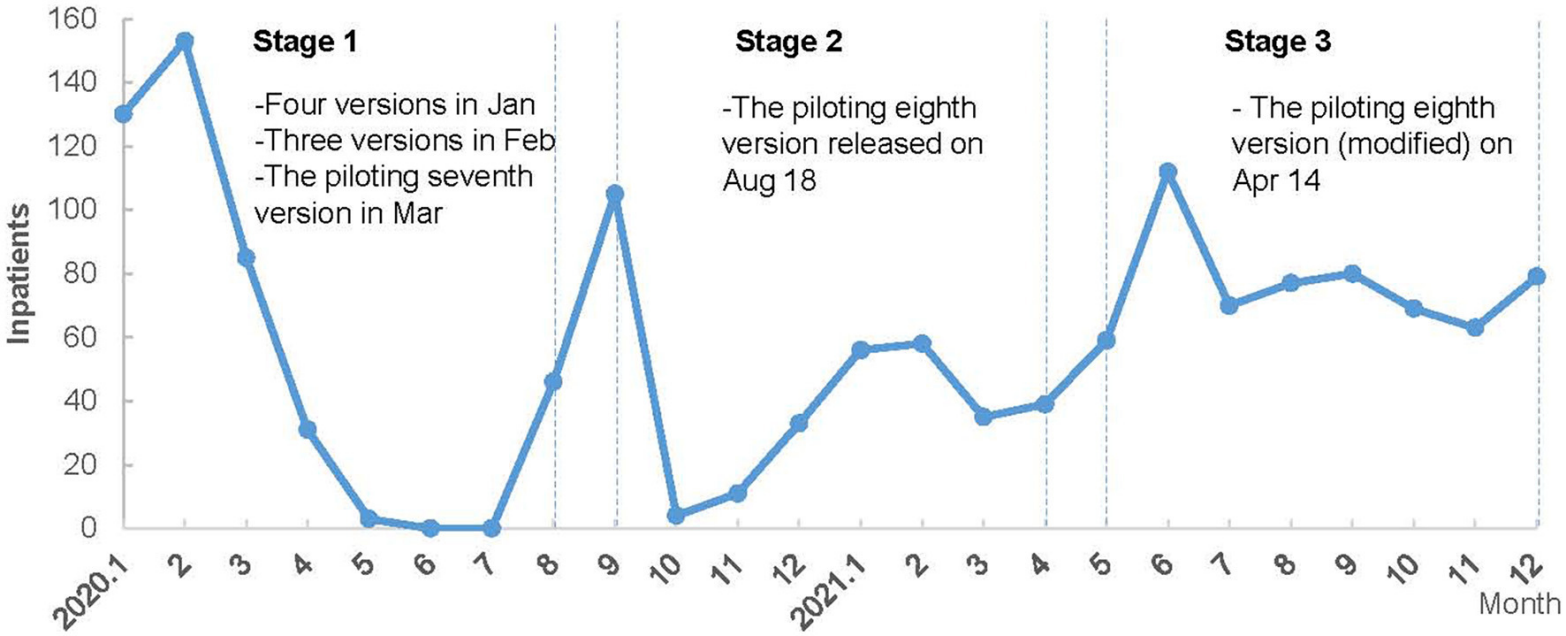
The monthly distribution of COVID-19 inpatients and clinical treatment guidelines in 2020 and 2021.

Considering disease evolution and knowledge update, combined with clinical and public health expert consultation (from the hospital and health department), we divided the study period into three stages to analyze the changing trend and difference of treatment cost among different stages for COVID-19 inpatients. Figure 1 shows the distribution of COVID-19 inpatients in 2020–2021 and the treatment guidelines used.

- Stage 1: from January 2020 to August 2020—the first COVID-19 diagnosis and treatment guideline was developed on January 15, 2020. Then, it was updated to the seventh version by March 2020 along with the gradually deeper understanding of this infectious disease. This stage could be regarded as piloting and exploring COVID-19 treatment.
- Stage 2: from September 2020 to April 2021—the eighth piloting version of COVID-19 diagnosis and treatment guideline were implemented which was built on the previous COVID treatment experiences and the clinical guidelines from WHO and other foreign countries.
- Stage 3: from May 2021 to December 2021—the piloting eighth version (modified) of COVID-19 diagnosis and treatment guideline has been implemented since April 2021 and guided clinical practices until the end of 2021. The clinical treatment guideline gradually achieved consensus and became standardized at this stage.

### 2.4. Data analysis

#### 2.4.1. Main outcome measures

The treatment cost for COVID-19 inpatients was measured in two ways: (a) mean value of treatment cost and specific cost components (medicine, lab testing, medical imaging, beds, consultation, nursing, medical therapy, medical materials and surgical cost); (b) percentage of specific cost component (%), defined by the proportion of this cost component accounted for total treatment cost.

#### 2.4.2. Statistical analysis

This study focused on the two key factors (i.e., COVID-19 clinical classifications and admission stages) affecting COVID-19 inpatient treatment cost. Therefore, all the comparisons regarding the COVID-19 inpatient treatment cost and cost components were made across seven COVID-19 clinical classifications and three admission stages (defined in Section 2.3) by descriptive statistics and multi-variable linear regression. Descriptive analysis was used to show the summary statistics of treatment cost and cost components of COVID-19 inpatients (mean, standard deviation, and percentage). The treatment cost in Chinese Renmibi (CNY) were changed into USD (annual exchange rate in 2021: USD 1.0 = CNY 6.45). Then, the multi-variable linear regression models were used by controlling inpatients' socio-demographic characteristics (age, sex, insurance status) and hospitalization characteristics (length of stay, and comorbidity), while dependent variables (different types of treatment cost) were log-transformed due to the skewed distribution. Considering the occurrence of zero value, all the dependent variables were plus 1 before the log transformation performed based on the previous literature (20). The original regression estimates were then transformed by exponential function when explain results. A *P*-value of <0.05 was considered statistically significant. The Stata 16 for Windows (Stata Corp, College Station, TX, USA) software was used for the statistical analysis.

Considering the readability, we first presented the descriptive summary of total treatment cost and cost components (Section 3.2), and then showed the regression results (Section 3.3).

## 3. Results

### 3.1. Characteristics of the sample

The sample included 1,398 COVID-19 inpatients from January 10, 2020 to December 31, 2021. The sample characteristics is shown in Table 1. Overall, the majority of included COVID-19 inpatients were aged 18–45 years (59.3%) and 45–65 years (33.3%) while 3.8% were older than 65 years. Many (68.8%) were male. The number of convalescent cases accounted for the largest proportion of all COVID-19 inpatients (42.7%).

**Table 1:** Sample characteristics.

**Characteristics**	**COVID-19 inpatients (*n* = 1,398)**
**COVID-19 clinical classifications**	
Asymptomatic	260 (18.6)
Mild	70 (5.0)
Moderate	326 (23.3)
Severe	60 (4.3)
Critical	16 (1.1)
Re-positive	69 (4.9)
Convalescent	597 (42.7)
**Admission stages**	
Stage 1: Jan 2020–Aug 2020	448 (32.1)
Stage 2: Sep 2020–Apr 2021	341 (24.4)
Stage 3: May 2021–Dec 2021	609 (43.6)
**Age (in years)**	
0-	51 (3.7)
18-	829 (59.3)
45-	465 (33.3)
>65	53 (3.8)
**Sex**	
Female	436 (31.2)
Male	962 (68.8)
**Insurance status**	
Non-local health insurance	880 (51.6)
Local health insurance	368 (21.6)
None	389 (22.8)
LOS (in days)^a^	19.7 (9.8)
Comorbidity^a^	1.7 (2.1)

LOS, length of stay; SD, standard deviation.

a The value is mean (SD).

### 3.2. Summary of total treatment cost and cost components for COVID-19 inpatients

#### 3.2.1. Total treatment cost

The summary of total treatment cost for COVID-19 inpatients is reported in Table 2. The treatment cost was USD 3,328.8 for all the included COVID-19 cases. The highest treatment cost occurred in the critical cases (USD 66,563.7), which was ~40 times of average cost of asymptomatic cases (USD 1,759.1). The re-positive cases incurred the least treatment cost (USD 1,038.0). The difference of treatment cost was not statistically significant across three admission stages for asymptomatic, mild, and severe cases, while a remarkable difference with statistical significance was shown for moderate, convalescent, and re-positive cases.

**Table 2:** Summary of total treatment cost for COVID-19 inpatients.

**COVID-19 clinical classifications**	**Total**		**Stage 1**		**Stage 2**		**Stage 3**		***P-*value^a^**
	***n***	**Mean (SD)**	***n***	**Mean (SD)**	***n***	**Mean (SD)**	***n***	**Mean (SD)**	
Asymptomatic	260	1,759.1 (1,014.2)	49	1,673.8 (735.0)	100	1,667.4 (858.9)	111	1,879.4 (1,223.4)	0.257
Mild	70	2,575.9 (1,036.7)	22	2,364.8 (914.9)	0	–	48	2,672.6 (1,083.2)	0.252
Moderate	326	3,782.0 (2,295.6)	219	3,622.7 (2,185.6)	25	5,260.9 (3,654.9)	82	3,756.4 (343.0)	0.003
Severe	60	12,052.3 (6,122.0)	56	12,046.5 (6,271.3)	0	–	4	12,133.4 (4,002.8)	0.978
Critical	16	66,563.7 (51,405.3)	15	69,260.8 (52,024.5)	1	26,105.8 (–)	0	–	–
Convalescent	597	1,546.5 (7,982.1)	86	3,644.7 (20,404.8)	206	1,018.8 (515.2)	305	1,311.3 (2,613.3)	0.028
Re-positive	69	1,038.0 (643.4)	1	2,046.2 (–)	9	1,635.9 (712.8)	59	929.7 (574.8)	0.002
Total	1,398	3,328.8 (10,480.7)	448	6,599.2 (17,784.7)	341	1,609.9 (2,070.6)	609	1,885.5 (2,414.6)	<0.001

SD, standard deviation; –, not applicable.

a ANOVA analysis.

#### 3.2.2. Cost components by clinical classifications

Table 3 shows the mean value of cost components and their percentage of total treatment cost for COVID-19 inpatients. For severe and critical cases, western medicine incurred the highest treatment cost, accounting for over 40% of total cost, followed by lab testing, medical therapy, and medical imaging cost. For the other five COVID-19 clinical classifications, lab testing cost accounted for the largest proportion of inpatient treatment cost, with a range of 32%−51%. Additionally, only critical cases occurred surgical cost with mean value of USD 745.9, which only accounted for 1.1% of total treatment cost.

**Table 3:** Cost components of COVID-19 inpatients by clinical classifications (mean, %)^a^.

**Cost components**	**All cases (*n* = 1,398)**	**Asymptomatic (*n* = 260)**	**Mild (*n* = 70)**	**Moderate (*n* = 326)**	**Severe (*n* = 60)**	**Critical^b^ (*n* = 16)**	**Re-positive (*n* = 69)**	**Convalescent (*n* = 597)**
Western medicine	935.3 (9.6)	94.8 (5.4)	277.3 (10.8)	848.3 (22.4)	5,226.1 (43.4)	32,093.0 (48.3)	42.3 (4.1)	262.2 (17.0)
Traditional medicine	41.4 (1.5)	23.5 (1.3)	60.4 (2.4)	60.3 (1.6)	119.1 (1.0)	451.9 (0.7)	14.9 (1.4)	20.8 (1.3)
Lab testing	1,093.8 (40.7)	898.6 (51.1)	1,305.4 (50.7)	1,552.7 (41.1)	3,146.9 (26.1)	11,555.0 (17.4)	413.0 (39.8)	495.3 (32.0)
Medical imaging	301.6 (11.1)	243.1 (13.8)	289.5 (11.2)	400.1 (10.6)	974.7 (8.1)	3,094.9 (4.7)	117.4 (11.3)	153.4 (9.9)
Beds	209.8 (13.7)	187.5 (10.7)	241.1 (9.4)	226.2 (6.0)	260.6 (2.2)	366.8 (0.6)	180.2 (17.4)	200.9 (13.0)
Consultation	154.4 (10.1)	140.0 (8.0)	164.7 (6.4)	160.4 (4.2)	216.9 (1.8)	345.6 (0.5)	136.6 (13.2)	146.9 (9.5)
Nursing	180.2 (9.4)	125.2 (7.1)	170.4 (6.6)	184.9 (4.9)	394.3 (3.3)	1,823.1 (2.7)	121.4 (11.7)	144.1 (9.3)
Medical therapy	292.4 (3.1)	33.1 (1.9)	55.3 (2.1)	297.1 (7.9)	1,416.9 (11.8)	10,042.6 (15.1)	8.2 (0.8)	89.1 (5.8)
Medical materials	109.1 (0.8)	11.3 (0.6)	9.9 (0.4)	50.1 (1.3)	295.4 (2.5)	6,014.0 (9.0)	2.5 (0.2)	31.0 (2.0)

a The values in parentheses are the percentage of this cost component accounted for total treatment cost (%).

b Only critical cases occurred surgical cost with mean value of USD 745.9 (accounting for 1.1% of total treatment cost) which was not listed in the table.

#### 3.2.3. Cost components by admission stages

The COVID-19 inpatient cost components varied across different admission stages as shown in Table 4. The largest cost was consumed on western medicine, which reached 38.5% at stage 1, while it accounted for ~10% of total treatment cost in the next two stages. Lab testing increased obviously and accounted for the largest proportion of cost in the admission stages 2 (38.6%) and 3 (42.7%). Constrained by sample size of different COVID-19 clinical classifications at different stages, here we further looked into the cost components of asymptomatic, moderate, and convalescent cases. The results show that the lab testing cost accounted for the largest proportion of total treatment cost at all the three stages and the percentage of western medicine reduced significantly from stage 1 to stage 3.

**Table 4:** Cost components of COVID-19 inpatients by admission stages (mean, %)^a^.

**Cost components**	**All cases (*****n*** = **1,398)**			**Asymptomatic (*****n*** = **260)**			**Moderate (*****n*** = **326)**			**Convalescent (*****n*** = **597)**		
	**Stage 1**	**Stage 2**	**Stage 3**	**Stage 1**	**Stage 2**	**Stage 3**	**Stage 1**	**Stage 2**	**Stage 3**	**Stage 1**	**Stage 2**	**Stage 3**
Western medicine	2,543.0 (38.5)	144.0 (8.9)	195.7 (10.4)	161.7 (8.3)	68.9 (3.7)	88.7 (2.6)	1,000.0 (21.3)	949.8 (12.0)	412.1 (8.5)	1,093.9 (11.0)	52.4 (3.4)	169.3 (2.8)
Traditional medicine	80.8 (1.2)	9.7 (0.6)	30.1 (1.6)	34.3 (2.0)	12.2 (0.5)	29.0 (1.1)	62.3 (1.7)	44.5 (0.9)	59.6 (1.5)	71.1 (4.5)	3.5 (0.3)	18.4 (1.6)
Lab testing	1,845.6 (28.0)	621.9 (38.6)	804.9 (42.7)	836.8 (51.5)	852.6 (53.7)	967.3 (51.4)	1,424.4 (43.1)	1,791.8 (37.1)	1,822.3 (50.1)	1,148.1 (45.3)	321.1 (32.3)	429.0 (33.9)
Medical imaging	468.6 (7.1)	223.7 (13.9)	222.2 (11.8)	218.4 (13.3)	253.8 (15.2)	244.3 (13.0)	332.9 (10.2)	669.8 (13.9)	497.1 (13.2)	280.8 (7.3)	138.1 (13.7)	127.8 (9.0)
Beds	206.6 (3.1)	190.9 (11.9)	222.6 (11.8)	149.1 (8.9)	172.1 (10.0)	218.3 (12.7)	203.2 (6.6)	288.7 (7.2)	268.6 (8.3)	191.2 (11.6)	189.4 (19.8)	211.3 (21.8)

a The values in parentheses are the percentage of this cost component accounted for total treatment cost (%).

### 3.3. Regression analysis of treatment cost and cost components of COVID-19 inpatients

#### 3.3.1. The underlying factors of COVID-19 inpatient treatment cost

The multi-variable linear regression results show that significant increases of treatment cost have been observed among mild cases (by 30.0%), moderate cases (by 49.2%), severe cases (by 228.7%), and critical cases (by 680.7%), while reductions have been shown among re-positive cases (by 43.1%) and convalescent cases (by 38.6%), by comparing with asymptomatic cases. Meanwhile, compared with stage 1, the treatment cost decreased significantly at stage 2 and stage 3 by 7.6 and 17.9% respectively. The other underlying factors of COVID-19 inpatient treatment cost are shown in the Supplementary Figure S2 and Supplementary Table S2.

#### 3.3.2. Key cost components across COVID-19 clinical classifications by regression

**Figure 2:**
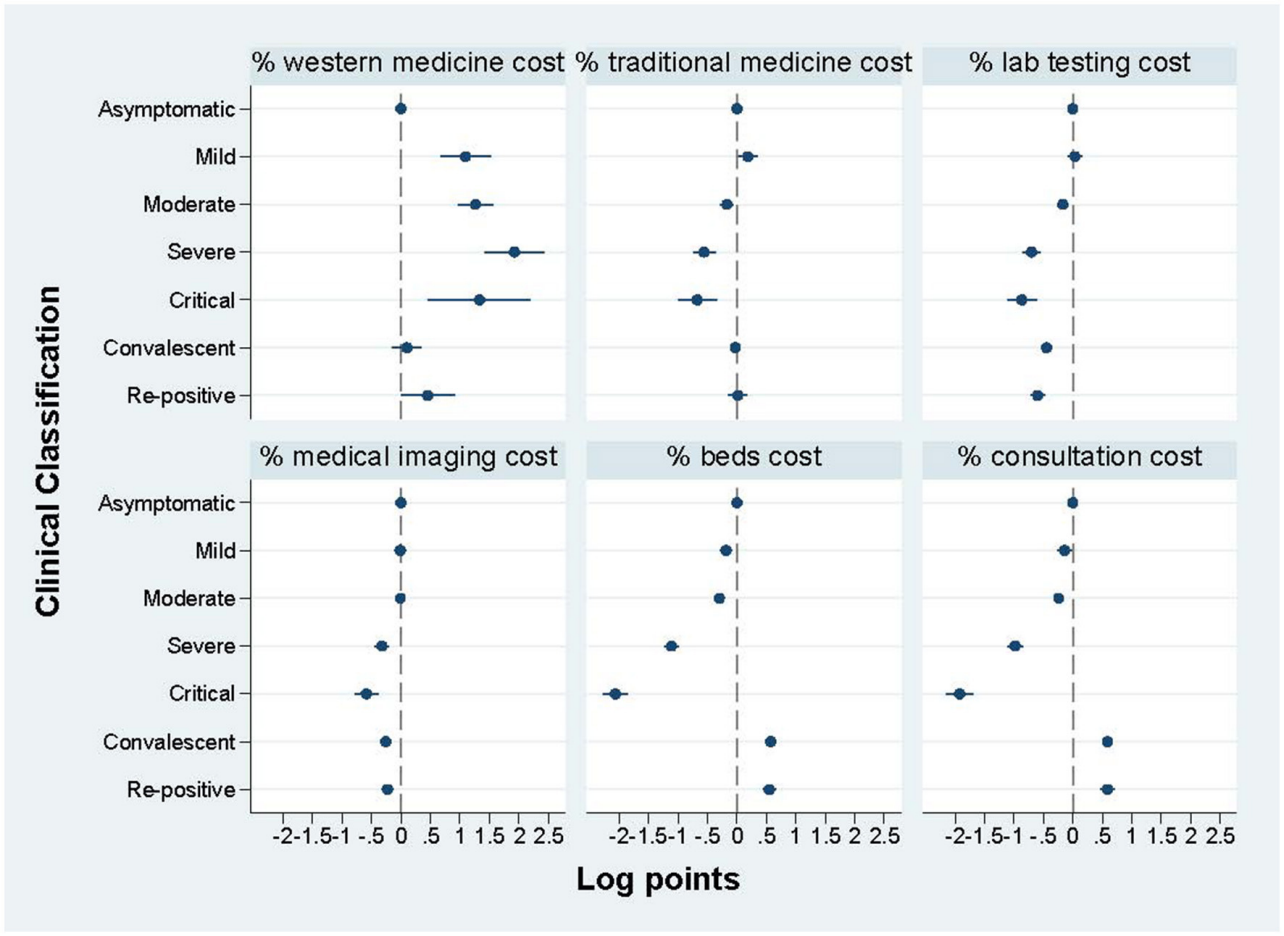
The percentage (%) of key cost components across clinical classifications by regression.

Figure 2 shows the regression results of the percentage of key cost components (% of total treatment cost) across seven clinical classifications. The following results are all based on a comparison with asymptomatic cases (reference group in the regression). The proportion accounted by western medicine cost in the other six clinical classifications is significantly larger, particularly for severe cases (by 5.9 times) and critical cases (by 2.8 times). Traditional medicine has been used more in mild cases with 20.2% increase. The use of lab tests was significantly reduced in severe cases by 50.3%, critical cases by 57.9%, convalescent cases by 36.1%, and re-positive cases by 44.9%. The largest reduction of medical imaging cost percentage was seen in critical cases by 44.5%. The cost of beds and consultation in convalescent and re-positive cases accounted for a higher proportion of total treatment cost while it shows distinct reduction in the other four cases. The regression table is shown in Supplementary Table S3.

#### 3.3.3. Key cost components across admission stages by regression

**Figure 3:**
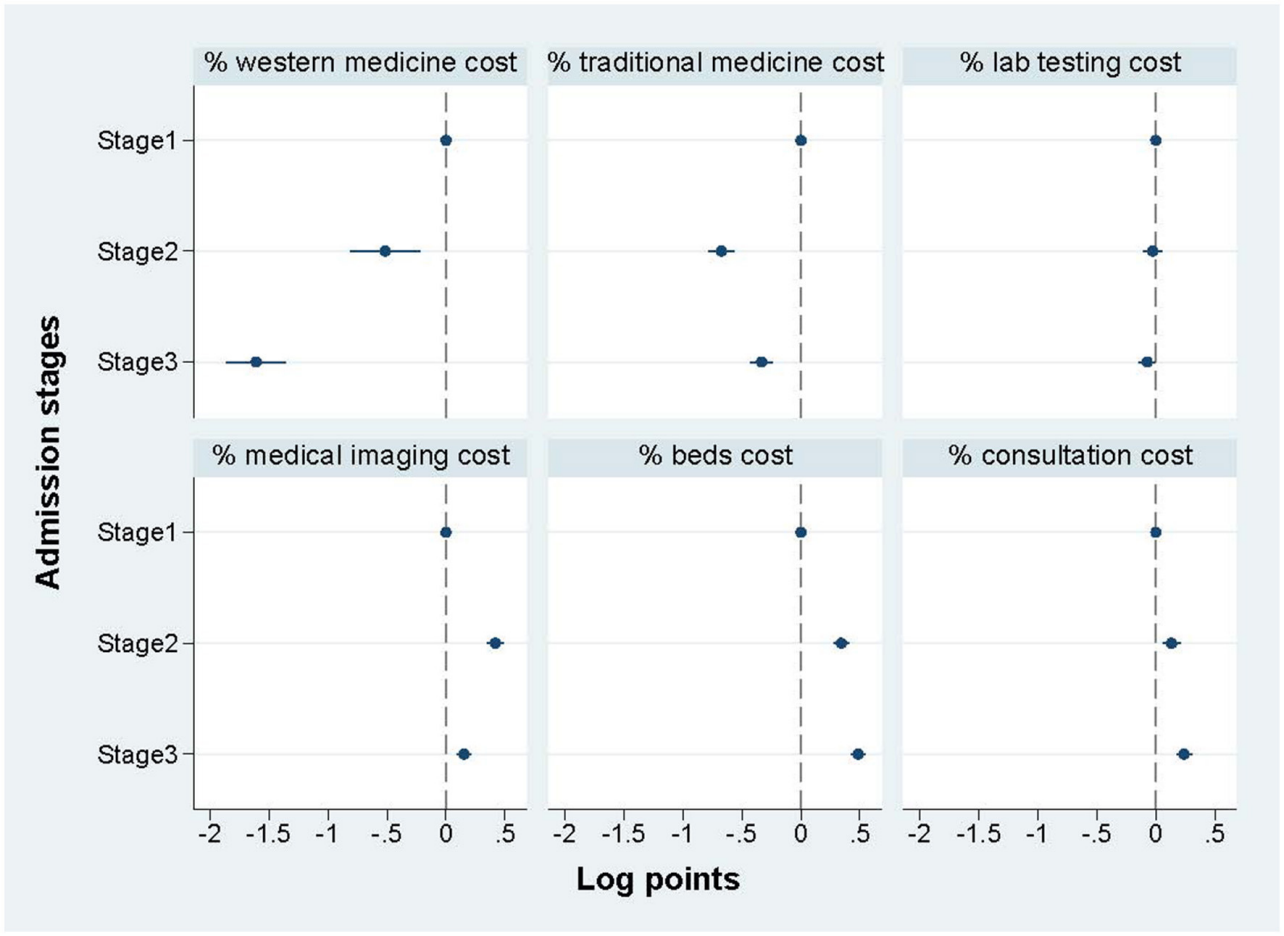
The percentage (%) of key cost components across admission stages by regression.

Figure 3 shows the regression results of the percentage of key cost components (% of total treatment cost) among three admission stages, and shows a distinct difference among the three stages. Specifically, compared with the COVID-19 cases at stage 1, the cases treated at stages 2 and 3 the following two stages spent much less proportion on medicine for both western medicine and traditional medicine, while the cases treated at stages 2 and 3 spent more proportion on medical imaging cost, beds cost, and consultation cost. The cost of lab testing showed no significant difference among stages. The regression table is shown in Supplementary Table S4.

## 4. Discussion

This study provides the latest comprehensive evidence based on real-word data, regarding total treatment cost and specific cost components of COVID-19 inpatients in 2020–2021 in Shenzhen, China. Our findings reveal that the treatment cost for COVID-19 inpatients varied across clinical classifications and admission stages. Compared with asymptomatic cases, the treatment cost significantly increased for critical, severe, moderate and mild cases while reductions were shown in re-positive and convalescent cases. For severe and critical cases, the highest proportion of treatment cost was spent on western medicine while lab testing cost accounted for the largest proportion for the other five COVID-19 clinical classifications. Compared with stage 1, the treatment cost decreased significantly at stage 2 and stage 3.

First of all, our study further confirmed the total treatment cost is positively connected with the severity of four clinical classifications of COVID-19 confirmed cases because the cost significantly increased from mild to critical cases. This finding is consistent with previous evidence regarding treatment cost of COVID-19 (16, 21, 22). More importantly, this study provides evidence of treatment cost of three special COVID-19 clinical classifications, that is, asymptomatic cases, convalescent cases, and re-positive cases, which has rarely been analyzed in current studies.

In particular, convalescent cases of COVID-19 inpatients accounted for 42.7% of total included inpatients and its treatment cost decreased by 38.6% compared with asymptomatic cases. The convalescent cases are relatively special for COVID-19 inpatients because it is more likely to be the quarantine stage in hospital, which is different from most countries outside of China. Convalescent cases have recovered from clinical symptoms related to COVID-19 and confirmed with negative nucleic acid testing results. From the perspectives of saving treatment cost, further discussion is warranted regarding whether or not convalescent cases should be treated in hospital. It is particularly unique that lab testing cost dominated in the treatment cost for convalescent cases. Moreover, relatively more cost has been spent on beds and consultation, which differentiated from the four clinical classifications of confirmed cases. It was closely linked with the clinical characteristics of convalescent cases because more attention has been paid to the lab testing cost to avoid the recurrence of COVID-19 and the treatment of comorbidities.

Second, the difference of the percentage of cost components accounted for total treatment cost reflects the treatment focus and the utilization of medical resources for different COVID-19 clinical classifications. This finding is particularly valuable because it indicates key supervision points along with gradually standardized treatment of COVID-19 inpatients. Taking some remarkable characteristics of cost components for example, the largest proportion (over 40%) was western medicine for severe and critical cases, which means that the rational use of western medicine should be paid more attention in the future. Moreover, we find that five COVID-19 clinical classifications (asymptomatic, mild, moderate, re-positive, and convalescent cases) spent the largest proportion on lab testing cost with the range of 32%−51% and is much higher in the asymptomatic cases after controlling related variables. Therefore, how to rationally use lab tests during the treatment of COVID-19 inpatients is vital, in order to achieve standardized treatment in the future.

Furthermore, in China, the cost of treating COVID-19 inpatients is fully covered by fiscal subsidy and the China Health Insurance Fund (13). This is different from most countries as there is no out-of-pocket payment for COVID-19 inpatients themselves in China (23). However, in the meanwhile, it poses huge economic burden on the health insurance fund and the Government. The variation of cost components provides us with major targets to be improved upon in the future, in order to achieve the best cost-effectiveness of treatment strategies for different COVID-19 clinical classifications. If future study could further examine the rationalization of lab testing and western medicine costs based on the findings of this study, it would greatly help save the health resources along with the regular prevention and control of COVID-19.

Third, timely clinical treatment guidelines are vital for the standardized treatment of COVID-19 inpatients. In China, eight versions (including modified versions) of COVID-19 clinical treatment guidelines were released during the study period. The regression results show that the decreasing trend of total cost has been observed at stages 2 and 3: it was suggestive that the reduction of total treatment cost positively correlated with the treatment guidelines. The development and update of clinical treatment guidelines are based on the timely clinical evidence and disease evolution, so this finding partly reflects the normalization of treating COVID-19 inpatients (18). Additionally, the increasing dose of COVID-19 vaccine and quick changing of variants of omicron are also two possible underlying factors affecting inpatient treatment cost for COVID-19 patients. The evidence has already shown that widespread vaccination could protect people from the virus and eliminate infectious symptoms (24, 25). In Shenzhen, there were 18,867,700 people who received the first dose and 17,719,100 people who received the full dose. The completion rate of booster immunization was 62.01% by the data on January 6, 2022 (26). Meanwhile, the symptoms infected by omicron variant has also been reported less severe compared with the original virus or delta variants in the early period of pandemic (27). Therefore, they also probably have contributed to the inpatient treatment cost reduction. The immunization data of COVID-19 inpatients was not available so we could not control this factor in this study. Moreover, the proportion of lab testing cost accounted for total treatment cost has shown no significant difference among the three stages. This finding further confirmed the vital role of lab testing during the treatment of COVID-19 inpatients and the need for strict supervision of its rational use.

Finally, compared with other relevant studies regarding treatment cost for COVID-19 inpatients, our estimate of USD 3,328.8 is much lower than the assessment of USD 6,825 by Li et al. (16), who used the data from 70 inpatients in Shandong province, China in January–March 2020. The cost components also show distinct differences: the medicine accounted for 45.1% of total treatment cost in Li's study while the lab test took account of the largest proportion (40.7%) in our study. We also find that our estimate is similar to the of USD 3,235 by Jin et al. (12), using the data from government reports and other publications during January–March 2020. We are not able to further compare the difference among clinical classifications and admission stages as both studies did not show specific details in this regard. The comparison with these two studies further supports the importance and the value of analyzing treatment cost for COVID-19 inpatients in a longer period and in a large sample to observe the reliable changing trend for policy making. Compared with similar studies in Spain and the USA, the absolute treatment cost in our study is much lower than the value USD 10,744 in Spain and the values of USD 11,267 (5)−43,986 (8) in the USA. Moreover, the COVID-19 treatment cost in our study took account of 17.2% of the GDP per capita adjusting by Purchasing Power Parity (PPP) while it was 26.4% in Spain and 16.3%−63.5% in the USA (28). Considering the out of pocket payment for COVID-19 in Spain and USA, the role of reimbursement policy in the difference of treatment cost for COVID-19 inpatients worth to be further explored. Due to huge variations in the hospital payment system, we could not further compare the difference among cost components in detail between China and other countries.

Our study has important strengths and policy implications. This study fills an important evidence gap by presenting comprehensive situation of treatment cost and related cost components for COVID-19 inpatients in 2020–2021 in China. It is rarely seen in previous work which mainly used macro-level data, or had a short study period and limited sample of COVID-19 inpatients. We identified the difference of inpatient treatment cost across seven COVID-19 clinical classifications and the changes at three admission stages. Our findings can be used in three aspects to shape suitable policy design for COVID-19 inpatients in the future: first, it informs the financial burden that the health insurance fund and the Government experience to form reimbursement policy in the next step; second, it helps concentrate on the essential points need to be noticed in the future COVID-19 inpatient treatment guidelines, that is, the rational use of western medicine in critical and severe cases and lab tests in the other five COVID-19 cases; third, it reminds of hospital administrators and policy makers the importance of suitable policy design for convalescent cases, which accounted for 43% of total cases with lab testing cost dominated in total treatment cost; finally, it is urgently needed to establish the organized hierarchical medical system for dealing with COVID-19 with different severities. Based on the treatment cost in the real world in our study, it would save substantial medical resources for the patients in need and to improve the sustainability and affordability of fiscal subsidy and medical insurance fund for keeping the less severe cases such as asymptomatic, mild and convalescent cases at home or primary health institutions instead of all admitting to the designated hospital Our findings partly supported the recent easing of zero-COVID policy in December 2022. The future policy design need to pay special attention on the health education for the public regarding COVID-19 prevention and treatment, and more importantly, to make the primary health institutions professionally equipped with essential medicines, lab testing and medical imaging equipment, whereas dominant proportion of treatment cost were found occurring in asymptomatic, mild and convalescent cases in this study.

Three limitations of this study should also be noticed. First, 401 cases were excluded due to lack of complement inpatient information such as clear COVID-19 clinical classifications and socio-demographic information, which may result in some bias on the distribution of sample characteristics. In particularly, nearly half of the excluded cases occurred in the early 2020 (*n* = 210) due to lack of COVID-19 clinical classifications (only labeled with COVID-19 without further categorization). It may cause underestimation in our study of the difference among admission stages. Second, COVID-19 evolved quickly to variants of delta and omicron, and the treatment plan and corresponding costs differentiated. The COVID-19 vaccination also plays an important role in the treatment cost for COVID-19 inpatients. Unfortunately, such data regarding the COVID-19 vaccination and the type of infected virus was not available at the micro level, which may lead to some bias for our regression estimations. Future studies could further analysis the impact of vaccination and different novel coronavirus on the hospitalized cost based on our findings. Third, the clinical guidelines have not been standardized in China although timely updates were released along with the disease evolution. Therefore, unavoidable trial or errors may inflate the treatment cost and result in the overestimates of the actual treatment cost during the pandemic period. Additionally, our study only focused on the direct medical cost for COVID-19 inpatients and indirect costs related to COVID-19 inpatient treatment were not considered in this study.

## 5. Conclusion

By using 2020–2021 data regarding actual COVID-19 inpatient treatment cost, our study shows that the treatment cost and cost components varied across different clinical classifications and admission stages in Shenzhen, China. Significant increases of treatment cost have been shown in the critical and severe cases, while distinctive reductions have been seen in re-positive and convalescent cases by comparing with asymptomatic cases. The largest proportion of inpatient treatment cost was spent on western medicine for severe and critical cases while lab testing cost played a dominant role in the other five COVID-19 clinical classifications. A decreasing trend of treatment cost was observed at stages 2 and 3 with the main drive from the reduction of western medicine cost. Our findings are particularly valuable to better inform policy makers and hospital administrators regarding future COVID-19 clinical treatment guidelines and prevention and control policy design, to achieve the best utilization of healthcare resources. We also recommend that more evidence is needed for the rational use of lab tests and western medicine cost in the COVID-19 treatment and suitable policy design for convalescent cases.

## Data availability statement

The data analyzed in this study is subject to the following licenses/restrictions. The datasets generated and/or analyzed during the current study are not publicly available due to the data management requirement but are available from the corresponding author on reasonable request Requests to access these datasets should be directed at: XW, wang.xiaowan@imicams.ac.cn.

## Author contributions

SY conceptualized study design, analyzed data, and drafted the manuscript. TL analyzed and interpreted the data and modified the manuscript. XW, CC, and LL provide advice on the analysis framework and critical revision of the manuscript. All authors have read and approved the final manuscript.
