# Supplementary material for: Treatment cost assessment for COVID-19 inpatients in Shenzhen, China 2020–2021: facts and suggestions
Source: Front Public Health. 2023 May 4;11:1066694. doi: 10.3389/fpubh.2023.1066694 (PMC10192705; doi:10.3389/fpubh.2023.1066694)
Supplement: Supplementary file 1 [file Data_Sheet_1.pdf]

Supplementary file

Table S1 Clinical classification of COVID-19 inpatients

**Clinical Classification**	**Characteristics**
Asymptomatic	Positive result for the nucleic acid test for SARS-CoV-2 without any clinical symptoms
Mild	The clinical symptoms are mild and there is no pneumonia manifestation in imaging
Moderate	With the above clinical manifestations, imaging showed pneumonia
Severe	Adults meet any one of the following:<br>a. Shortness of breath, respiratory rate (RR) ≥ 30 times/min;<br>b. In the resting state, the pulse oxygen saturation (SpO2) is ≤93% while breathing ambient air;<br>c. Arterial partial pressure of oxygen (PaO2)/the fraction of inspired oxygen (FiO2) ≤300 mmHg (1 mmHg =0.133 kPa);<br>In areas with high altitudes (more than 1000m above sea level), PaO2/FiO2 should be adjusted according to the following formula: PaO2/FiO2 × (760/atmospheric pressure [mmHg]).<br>d. The clinical symptoms are progressively worse, and lung imaging shows that the lesion has progressed significantly >50% within 24–48 h.<br>Children meeting any of the following:<br>a. High fever lasting more than 3 days;<br>b. Shortness of breath (<2 months old, RR ≥ 60 beats/min; 2–12 months old, RR ≥ 50 beats/min; 1–5 years old, RR ≥ 40 beats/min; >5 years old, RR ≥30 times/min), the influence of fever and crying excluded;<br>c. In the resting state, the pulse oxygen saturation is ≤93% while breathing ambient air;<br>d. Respiratory distress (nostril flapping, three concave signs);<br>e. Drowsiness, convulsions;<br>f. Refusal to feed or feeding difficulties with signs of dehydration.
Critical	Meet any one of the following conditions:<br>a. Respiratory failure and mechanical ventilation;<br>b. Shock;<br>c. Intensive care unit (ICU) admission due to other organ failures.
Convalescent	a. Negative result for the nucleic acid test for SARS-CoV-2 without any clinical symptoms after hospitalised treatment;<br>b. Be required to stay in hospital for clinical observation in case of re-positive.
Re-positive	Be re-infected after discharged from hospital.

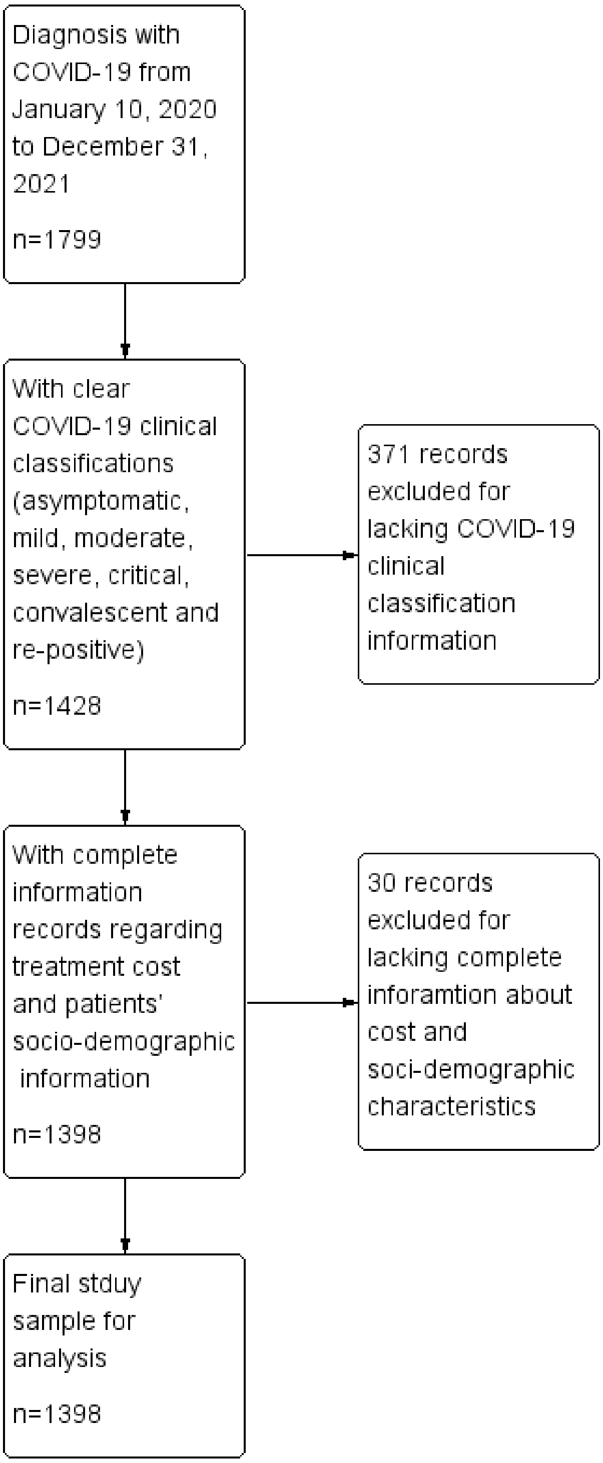

Figure S1 Sampling diagram

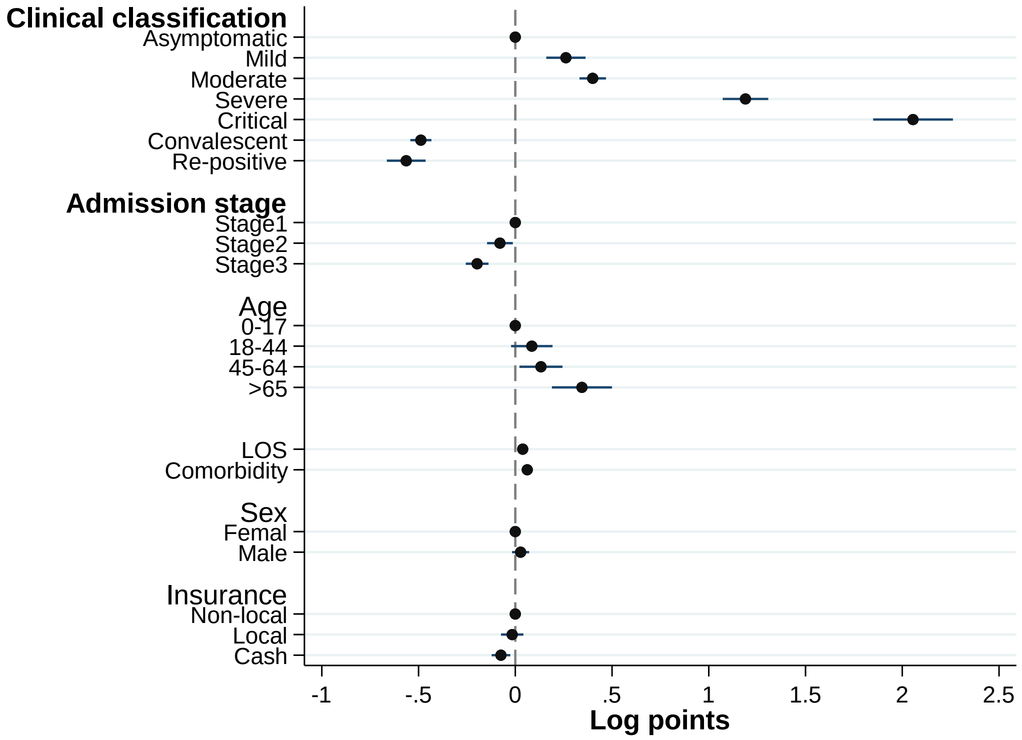

Figure S2 The underlying factors of COVID-19 inpatient treatment cost by regression analysis

Table S2 Regression results of the underlying factors of COVID-19 inpatient treatment cost

Log (treatment cost)	**Coef.**	**Std. Err.**	***P***	***95% Conf. Interval***	
COVID 19 clinical classifications (reference group: Asymptomatic)					
Mild	0.262	0.052	0.000	0.160	0.363
Moderate	0.400	0.035	0.000	0.332	0.469
Severe	1.190	0.060	0.000	1.072	1.307
Critical	2.055	0.105	0.000	1.849	2.262
Convalescent	-0.488	0.028	0.000	-0.543	-0.433
Re-positive	-0.564	0.051	0.000	-0.664	-0.463
Admission stages (Reference group: Stage 1)					
Stage 2	-0.079	0.034	0.020	-0.146	-0.013
Stage 3	-0.197	0.030	0.000	-0.256	-0.138
Age					
18-44	0.085	0.055	0.118	-0.022	0.193
45-64	0.133	0.057	0.019	0.021	0.244
>65	0.344	0.079	0.000	0.189	0.500
LOS	0.038	0.001	0.000	0.036	0.041
Comorbidity	0.062	0.005	0.000	0.052	0.072
Sex (Reference group: female)					
Male	0.027	0.023	0.224	-0.017	0.072
Insurance (Reference group: non-local)					
Local	-0.016	0.029	0.589	-0.074	0.042
None	-0.074	0.025	0.003	-0.122	-0.026

Table S3 Regression results of key cost components by COVID-19 clinical classifications

% of Key cost components[1]	COVID 19 clinical classifications (reference group: Asymptomatic)	Coef.	Std. Err.	*P*	*95% Conf. Interval*	
% western medicine cost	Mild	1.094	0.218	0.000	0.666	1.522
	Moderate	1.266	0.149	0.000	0.974	1.558
	Severe	1.927	0.253	0.000	1.431	2.423
	Critical	1.333	0.440	0.003	0.469	2.196
	Convalescent	0.100	0.126	0.426	-0.147	0.347
	Re-positive	0.452	0.231	0.050	-0.001	0.905
% traditional medicine cost	Mild	0.184	0.082	0.024	0.024	0.344
	Moderate	-0.167	0.055	0.002	-0.275	-0.059
	Severe	-0.557	0.095	0.000	-0.742	-0.371
	Critical	-0.672	0.166	0.000	-0.997	-0.348
	Convalescent	-0.026	0.044	0.549	-0.112	0.060
	Re-positive	0.014	0.081	0.859	-0.144	0.173
% lab testing cost	Mild	0.034	0.062	0.587	-0.088	0.156
	Moderate	-0.171	0.042	0.000	-0.253	-0.088
	Severe	-0.700	0.072	0.000	-0.842	-0.558
	Critical	-0.865	0.127	0.000	-1.114	-0.617
	Convalescent	-0.448	0.034	0.000	-0.514	-0.383
	Re-positive	-0.596	0.062	0.000	-0.717	-0.475
% medical imaging cost	Mild	-0.011	0.050	0.820	-0.109	0.086
	Moderate	-0.011	0.034	0.751	-0.077	0.056
	Severe	-0.322	0.058	0.000	-0.436	-0.208
	Critical	-0.589	0.101	0.000	-0.788	-0.390
	Convalescent	-0.258	0.027	0.000	-0.311	-0.204
	Re-positive	-0.227	0.053	0.000	-0.332	-0.122
% beds cost	Mild	-0.185	0.052	0.000	-0.288	-0.083
	Moderate	-0.298	0.035	0.000	-0.367	-0.229
	Severe	-1.112	0.061	0.000	-1.231	-0.992
	Critical	-2.061	0.107	0.000	-2.270	-1.852
	Convalescent	0.574	0.028	0.000	0.519	0.630
	Re-positive	0.551	0.052	0.000	0.449	0.653
% consultation cost	Mild	-0.141	0.056	0.013	-0.251	-0.030
	Moderate	-0.239	0.038	0.000	-0.314	-0.165
	Severe	-0.980	0.065	0.000	-1.107	-0.852
	Critical	-1.923	0.114	0.000	-2.146	-1.699
	Convalescent	0.586	0.030	0.000	0.527	0.645
	Re-positive	0.587	0.056	0.000	0.478	0.697

Note: [1] the dependent variable here is log transformed. To avoid the missing values, the calculation for this log transformed is "log (% of key cost components + 1)".

Table S4 Regression results of key cost components by admission stages

**% of Key cost components**	**Admission stages (Reference group: Stage 1)**	**Coef.**	**Std. Err.**	***P***	***95% Conf. Interval***	
% western medicine cost	Stage 2	-0.516	0.149	0.001	-0.809	-0.223
	Stage 3	-1.612	0.130	0.000	-1.866	-1.358
% traditional medicine cost	Stage 2	-0.676	0.054	0.000	-0.781	-0.571
	Stage 3	-0.334	0.047	0.000	-0.427	-0.241
% lab testing cost	Stage 2	-0.028	0.041	0.502	-0.108	0.053
	Stage 3	-0.074	0.036	0.041	-0.145	-0.003
% medical imaging cost	Stage 2	0.418	0.033	0.000	0.352	0.483
	Stage 3	0.153	0.030	0.000	0.095	0.211
% beds cost	Stage 2	0.342	0.034	0.000	0.274	0.410
	Stage 3	0.486	0.030	0.000	0.426	0.545
% consultation cost	Stage 2	0.133	0.037	0.000	0.061	0.206
	Stage 3	0.240	0.033	0.000	0.176	0.304

Note: [1] the dependent variable here is log transformed. To avoid the missing values, the calculation for this log transformed is "log (% of key cost components + 1)".
